# Unexpected Efficacy of a Novel Sodium Channel Modulator in Dravet Syndrome

**DOI:** 10.1038/s41598-017-01851-9

**Published:** 2017-05-10

**Authors:** Lyndsey L. Anderson, Nicole A. Hawkins, Christopher H. Thompson, Jennifer A. Kearney, Alfred L. George

**Affiliations:** Department of Pharmacology, Northwestern University Feinberg School of Medicine, Chicago, IL USA

## Abstract

Dravet syndrome, an epileptic encephalopathy affecting children, largely results from heterozygous loss-of-function mutations in the brain voltage-gated sodium channel gene *SCN1A*. Heterozygous *Scn1a* knockout (*Scn1a* ^+/−^) mice recapitulate the severe epilepsy phenotype of Dravet syndrome and are an accepted animal model. Because clinical observations suggest conventional sodium channel blocking antiepileptic drugs may worsen the disease, we predicted the phenotype of *Scn1a* ^+/−^ mice would be exacerbated by GS967, a potent, unconventional sodium channel blocker. Unexpectedly, GS967 significantly improved survival of *Scn1a* ^+/−^ mice and suppressed spontaneous seizures. By contrast, lamotrigine exacerbated the seizure phenotype. Electrophysiological recordings of acutely dissociated neurons revealed that chronic GS967-treatment had no impact on evoked action potential firing frequency of interneurons, but did suppress aberrant spontaneous firing of pyramidal neurons and was associated with significantly lower sodium current density. Lamotrigine had no effects on neuronal excitability of either neuron subtype. Additionally, chronically GS967-treated *Scn1a* ^+/−^ mice exhibited normalized pyramidal neuron sodium current density and reduced hippocampal Na_V_1.6 protein levels, whereas lamotrigine treatment had no effect on either pyramidal neuron sodium current or hippocampal Na_V_1.6 levels. Our findings demonstrate unexpected efficacy of a novel sodium channel blocker in Dravet syndrome and suggest a potential mechanism involving a secondary change in Na_V_1.6.

## Introduction

Epilepsy is one of the most common neurological disorders, with a lifetime incidence of 1 in 26. Approximately two-thirds of epilepsy has a substantial genetic component to its etiology. Channelopathies, particularly those involving voltage-gated sodium (Na_V_) channel genes such as *SCN1A*, are frequent causes of monogenic epilepsy^1–4^. *SCN1A* mutations result in a wide spectrum of epilepsy phenotypes ranging from simple febrile seizures to Dravet syndrome, a severe epileptic encephalopathy^5–9^. Dravet syndrome typically begins during the first year of life with generalized tonic-clonic or hemiclonic seizures, often precipitated by fever. Children with Dravet syndrome subsequently develop other seizures types and comorbidities, including cognitive impairment, ataxia and psychomotor dysfunction. They also respond poorly to currently available antiepileptic drugs and exhibit unfavorable long-term survival. More than 80% of Dravet syndrome patients have *de novo* heterozygous missense and truncation mutations in *SCN1A*, suggesting haploinsufficiency of *SCN1A* as the genetic cause^10^. Consistent with *SCN1A* haploinsufficiency in Dravet syndrome are clinical observations suggesting that conventional sodium channel blockers are ineffective and may even exacerbate the disease^7, 11, 12^.

Mice with heterozygous deletion of *Scn1a* (*Scn1a* ^+/−^) recapitulate many features of Dravet syndrome, including spontaneous seizures, hyperthermia-induced seizures and premature death^13–15^. Previous studies of *Scn1a* ^+/−^ mice have identified reduced sodium current density and impaired neuronal excitability in GABAergic interneurons leading to the prevailing hypothesis that impaired GABA-mediated inhibition is responsible for epileptogenesis in Dravet syndrome^13, 14, 16^.

Here we evaluated the effect of GS967 in the *Scn1a* ^+/−^ mouse model of Dravet syndrome. GS967 has been shown to preferentially inhibit persistent sodium current mediated by the cardiac voltage-gated sodium channel^17, 18^. GS967 has a long plasma half-life, excellent bioavailability, excellent brain penetration and is not metabolized^17, 19^. Recently, we have shown that GS967 exhibits antiepileptic activity in transgenic mice expressing a gain-of-function *Scn2a* mutation^20^. We hypothesized initially that GS967 would exacerbate the phenotype of *Scn1a* ^+/−^ mice. Unexpectedly, we found that GS967 treatment greatly improved survival and significantly lowered spontaneous seizure frequency in *Scn1a* ^+/−^ mice. These effects are explained by actions of GS967 on neuronal excitability and sodium current along with reduced protein levels of the Na_V_1.6 sodium channel.

## Results

### GS967 improves survival of *Scn1a*^+/−^ mice

Premature mortality in Dravet syndrome can be modeled in *Scn1a* ^+/−^ mice on a [C57BL/6 J x 129S6/SvEvTac]F1 genetic background. Most *Scn1a* ^+/−^ mice die between the third and fourth postnatal week of life with approximately 20% surviving to 8 weeks of age. We hypothesized that exposure to the novel sodium channel blocker GS967 would further accelerate premature death in *Scn1a* ^+/−^ mice in a manner consistent with reports that sodium channel blocking antiepileptic drugs aggravate Dravet syndrome^11, 12^. To test this hypothesis, mice were maintained on chow containing GS967 beginning at postnatal day 18 (P18) and survival was monitored until 8 weeks of age. We previously demonstrated that plasma and brain levels of GS967 sufficient to modulate brain sodium channels can be achieved with chronic oral administration^20^.

**Figure 1:**
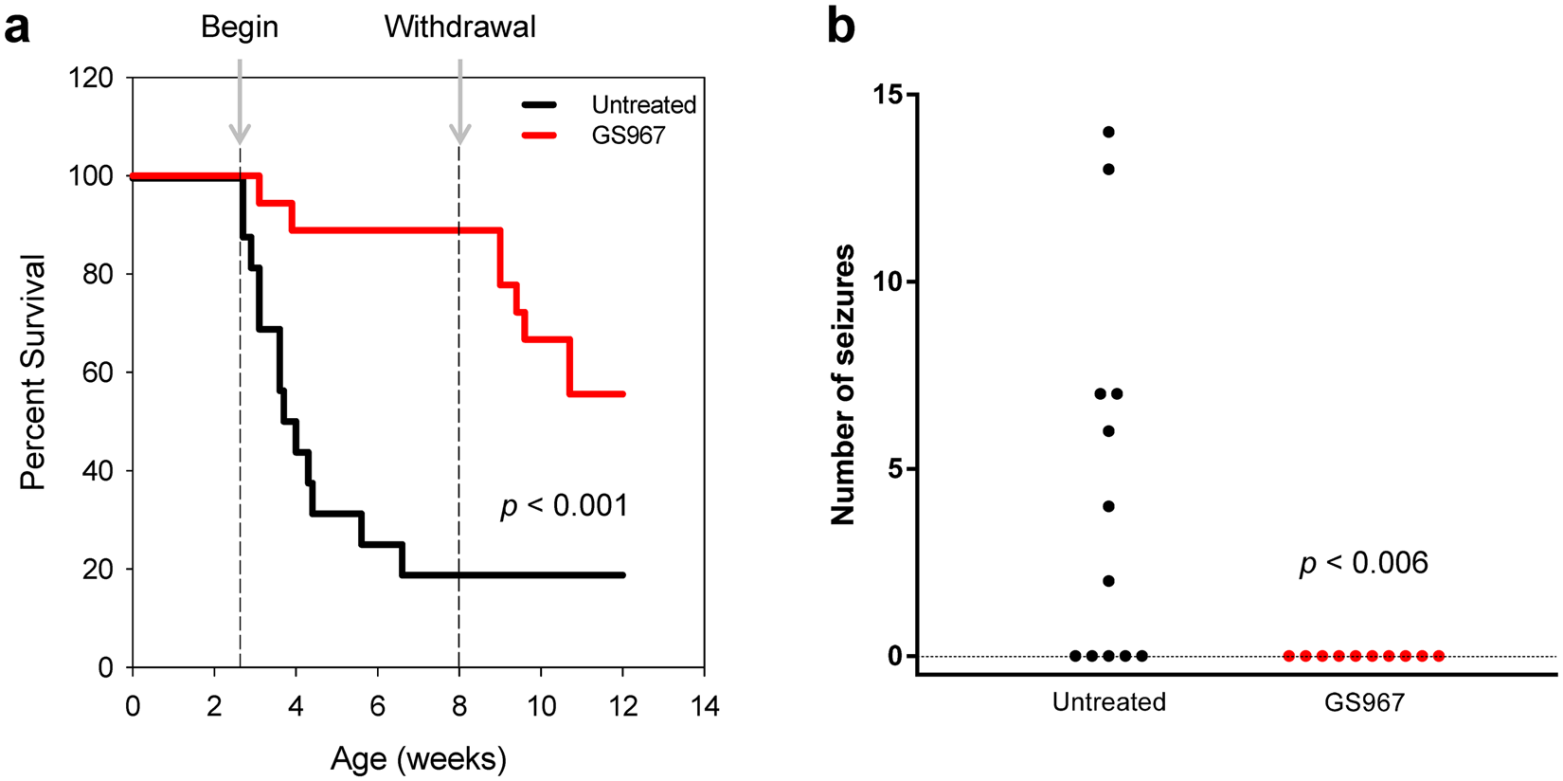
GS967 improved survival and reduced seizure frequency of *Scn1a* ^+/−^ mice. (**a**) Survival curves comparing untreated and GS967-treated *Scn1a* ^+/−^ mice. Treatment commenced at P18 (first dashed line) and was withdrawn at 8 weeks (second dashed line), with n = 18–37 mice per group. Survival difference between groups was significant (*p* < 0.001; Mantel-Cox log-rank test). (**b**) Number of seizures in 48 hours observed for untreated and GS967-treated *Scn1a* ^+/−^ mice (n = 10–12 mice per group) was significantly different (*p* < 0.006; non-parametric Mann-Whitney test).

Unexpectedly, *Scn1a* ^+/−^ mice treated with GS967 survived significantly longer than untreated *Scn1a* ^+/−^ littermates. Specifically, 90% of GS967-treated *Scn1a* ^+/−^ mice were alive at 8 weeks compared to 20% survival of untreated animals (*p* < 0.001, Fig. 1a). The survival advantage of treated animals was dependent upon continuous GS967 treatment, and withdrawal of GS967 treatment at 8 weeks was associated with a decline in survival over the ensuing 4 weeks (Fig. 1a).

### GS967 reduces seizure frequency in *Scn1a*^+/−^ mice

Because GS967 unexpectedly improved survival of *Scn1a* ^+/−^ mice, we sought to determine if this correlated with an antiepileptic action of the compound. *Scn1a* ^+/−^ mice have spontaneous generalized tonic-clonic seizures observable as early as P16 but becoming much more frequent by age P21–25. We initiated GS967 treatment in a cohort of *Scn1a* ^+/−^ mice at age P18 then compared the frequency of spontaneous seizures between treated and untreated mice at age P23–24, which corresponds to the period of highest seizure incidence with minimal animal loss from premature death. Average concentrations of GS967 in plasma and brain achieved in these mice were 1.0 ± 0.08 μM and 1.66 ± 0.11 μM, respectively. As a group, GS967-treated *Scn1a* ^+/−^ mice (n = 10) had no seizures over the 48 hour observation period compared to a total of 52 seizures in untreated mice (n = 12; p < 0.006, Fig. 1b). Quantifying seizure frequency over a longer time period also demonstrated that GS967-treated mice had a significantly lower seizure burden than untreated animals (GS967-treated: 3 seizures over 1065 hours, range 52.5–96 hours per animal, average 88.75, n = 12; untreated: 75 seizures over 958 hours, range 19–96 hours per animal, average 68.5, n = 14). The number of behavioral seizures was highly correlated with the number of electrographic seizures in both untreated and treated animals (see Methods). These findings indicated that GS967 exerted an anti-seizure effect in Dravet syndrome mice and this effect correlated with improved survival. Interestingly, GS967 had no effect on the temperature threshold of hyperthermia-induced seizures in *Scn1a* ^+/−^ mice (Supplemental Fig. S1).

By contrast, lamotrigine, a widely used sodium channel blocking antiepileptic drug, caused *Scn1a* ^+/−^ mice to exhibit a higher seizure frequency consistent with observations made in human Dravet syndrome^12^. Specifically, *Scn1a* ^+/−^ mice treated with lamotrigine (20 mg/kg/day) beginning at age P18 exhibited significantly more spontaneous seizures during a video monitored period between P21 and P25 (223 seizures over 863 hours, n = 9) than control animals (75 seizures over 958 hours, n = 14, *p* = 0.0047). Steady-state total plasma lamotrigine levels measured at the end of the experiment (3.5 µg/mL) were within the human therapeutic range (2.6–15 µg/mL)^21^.

### Effects of GS967 on neuronal excitability

A cellular mechanism implicated in Dravet syndrome is impaired GABAergic interneuron excitability^13, 14, 22, 23^. We have also observed an unexplained enhancement of sodium current and high frequency spontaneous action potential firing in excitatory pyramidal neurons emerging after age P21 that correlates with the age-dependent onset of seizures and accelerated mortality^16^. To investigate the effects on neuron excitability, we compared action potential firing frequencies among untreated, GS967-treated and lamotrigine-treated *Scn1a* ^+/−^ mice.

**Figure 2:**
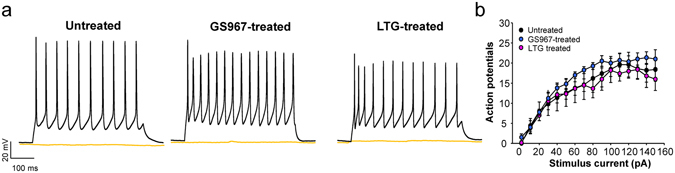
GS967 treatment of *Scn1a* ^+/−^ mice does not affect bipolar neuron excitability. (**a**) Representative action potentials in response to either 0 pA (orange traces) or 50 pA stimulus (black traces) recorded from bipolar neurons acutely isolated from untreated (*left*), GS967-treated (*middle*) and lamotrigine (LTG)-treated (right) *Scn1a* ^+/−^ mice. (**b**) Summary data plotting number of action potentials against stimulus current. Error bars represent SEM, with n = 5–7 per group. There were no significant differences among the groups at any stimulus current level.

Figure 2 illustrates action potential recordings from bipolar interneurons acutely isolated from untreated, GS967-treated and lamotrigine-treated *Scn1a* ^+/−^ mice (age range P21–24). None of these cell preparations exhibited spontaneous firing, and all had levels of evoked action potentials that were not significantly different from one another across a wide range of stimulus current amplitudes. These findings do not explain the observed anticonvulsant effects of GS967 nor the enhanced seizure phenotype associated with lamotrigine.

**Figure 3:**
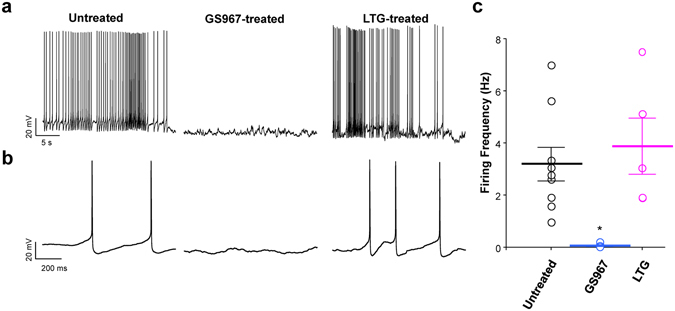
GS967 suppresses spontaneous firing of *Scn1a* ^+/−^ pyramidal neurons. (**a**) Representative spontaneous action potentials recorded from single pyramidal neuron acute isolated from untreated (*left*), (GS967-treated *Scn1a* ^+/−^ (*middle*) and LTG-treated (right) *Scn1a* ^+/−^ mice. Membrane potential was clamped at −80 mV, and spontaneous action potentials were recorded from *Scn1a* ^+/−^ neurons. (**b**) Expansion of the first 1 second shown in panel A for untreated (*left*), (GS967-treated *Scn1a* ^+/−^ (*middle*) and LTG-treated (*right*) *Scn1a* ^+/−^ mice (**c**) Scatter plot of spontaneous firing frequency. Individual cells are depicted as open circles and average firing frequencies are depicted by bars. Error bars represent standard error of the mean (SEM), with n = 5–9 cells per group (*p < 0.01 for comparison with untreated mice; one-way ANOVA followed by Tukey’s test).

By contrast, the electrophysiological properties of pyramidal neurons acutely isolated from untreated and GS967-treated *Scn1a* ^+/−^ mice (age range P21–24) were dramatically different (Fig. 3). Neurons from untreated *Scn1a* ^+/−^ mice exhibited high frequency spontaneous firing consistent with our previous observations^16^, whereas neurons from GS967-treated *Scn1a* ^+/−^ mice had no spontaneous firing. Pyramidal neurons from lamotrigine-treated *Scn1a* ^+/−^ mice exhibited firing frequencies that were not different from untreated animals. These findings correlate with the observed anticonvulsant effects of GS967 and the lack of efficacy observed for lamotrigine in *Scn1a* ^+/−^ mice.

To determine if a residual effect of GS967 on neurons following acute isolation was responsible for the absence of spontaneous firing, we measured the amount of sodium current remaining after a 100 ms inactivating prepulse, which evoked a significant difference in sodium current availability during acute exposure to GS967 by virtue of enhanced entry into and impaired recovery from inactivation (Supplemental Fig. S2). We observed no differences in normalized current remaining among wildtype, *Scn1a* ^+/−^ or GS967-treated *Scn1a* ^+/−^ mice following this inactivating pulse (0.87 ± 0.02, 0.89 ± 0.01, 0.88 ± 0.01, respectively), indicating there was no residual effect of GS967 on neuronal sodium channels under our experimental conditions. These data suggest that chronic GS967 exposure dampens pyramidal neuron sodium current density and excitability and these effects are uncoupled from the acute actions of the compound on sodium channels.

**Figure 4:**
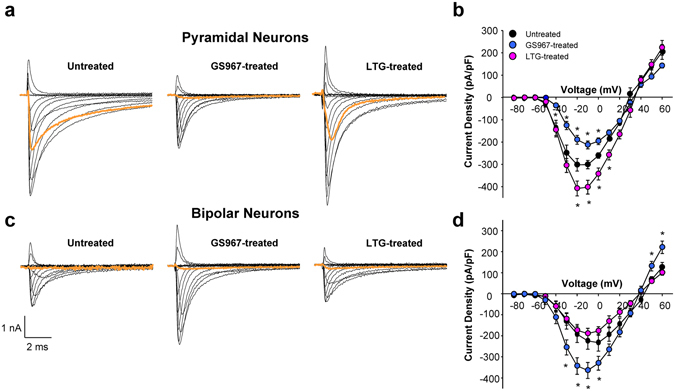
Chronic GS967 treatment of *Scn1a* ^+/−^ mice alters neuronal sodium current density. Representative traces of whole-cell sodium current from (**a**) pyramidal neurons or (**c**) bipolar neurons from untreated (*left*), GS967-treated (*middle*) and LGT-treated (*right*) *Scn1a* ^+/−^ mice. Peak sodium current density (normalized to cell capacitance) at tested potentials from (**b**) pyramidal neurons or (**d**) bipolar neurons from treated and untreated *Scn1a* ^+/−^ mice. Orange traces represent sodium current elicited by a −40 mV tested pulse, near the action potential threshold potential. Error bars represent SEM, with n = 7–16 per group (*p < 0.05 for comparison with untreated mice; one-way ANOVA followed by Tukey’s test).

Sodium current density in neurons isolated from untreated, GS967-treated and lamotrigine-treated *Scn1a* ^+/−^ mice was also different. Compared to cells from untreated mice, GS967 treatment was associated with significantly lower sodium current density (Fig. 4a,b) and a depolarized conductance-voltage relationship (Supplemental Fig. S3a) in pyramidal neurons. By contrast, sodium current density in pyramidal neurons from lamotrigine-treated mice was significantly greater than untreated animals (Fig. 4a,b). Opposite effects were observed in bipolar neurons; GS967-treatment was associated with greater peak sodium current density, whereas lamotrigine had no effect on sodium current density (Fig. 4c,d), but importantly there were no differences in sodium current density elicited at voltages near the action potential threshold (orange traces, Fig. 4c). Further, neither compound significantly affected conductance-voltage relationships in these neurons (Supplemental Fig. S3b). The divergent effects of GS967 and lamotrigine on neuronal sodium current density are correlated with the anticonvulsant effects of GS967 and the lack of efficacy of lamotrigine, respectively.

### GS967 reduces Na_v_1.6 protein levels

**Figure 5:**
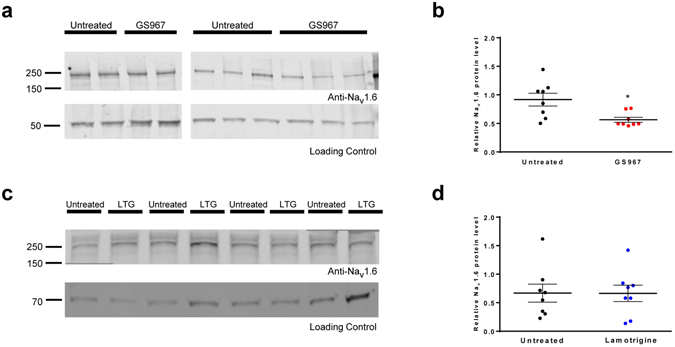
GS967 reduces neuronal Na_V_1.6 expression in *Scn1a* ^+/−^ mice. (**a**) Western blot analysis of Na_v_1.6 protein levels in hippocampal membrane preparations from *Scn1a* ^+/−^ mice. Representative blots of five biological replicates are shown for untreated and GS967-treated mice. (**b**) Scatter plot of densitometric analysis of Western blot data. Data points represent the average densitometry (≥2 technical replicates) for individual untreated or GS967-treated mice. The average densitometry values are depicted by the thick black line. Error bars represent SEM, with n = 2–8 mice per group (*p < 0.016; Student’s t-test). (**c**) Western blot analysis of Na_V_1.6 protein levels in hippocampal membrane preparations from *Scn1a* ^+/−^ mice. Representative blot from four biological replicates are shown for untreated and lamotrigine-treated mice. (**d**) Scatter plot of densitometric analysis of Western blot data. Data points represent the individual densitometry for untreated or lamotrigine-treated (LTG) mice. The average densitometry values are depicted by the thick black line. Error bars represent SEM, with n = 8 per treatment. There is no significant difference between groups. Images were cropped to improve conciseness and full length western blot images are presented in Supplemental Fig. S7.

A plausible explanation for normalization of pyramidal neuron sodium current density in *Scn1a* ^+/−^ mice chronically treated with GS967 is a change in the levels of sodium channel proteins. To investigate this possibility, we isolated hippocampi from untreated *Scn1a* ^+/−^ and chronically GS967 or lamotrigine-treated *Scn1a* ^+/−^ mice then measured total protein levels of the major brain sodium channels (Na_V_1.1, Na_V_1.2 and Na_v_1.6) using Western blot analysis. Protein levels of Na_V_1.1 and Na_V_1.2 were not significantly different between GS967-treated and untreated mice (Na_V_1.1, *p* = 0.299, n = 8–9 mice per treatment group; Na_V_1.2, *p* = *0.745*, n = 6–8 mice per treatment group; Supplemental Fig. S4). By contrast, Na_v_1.6 protein levels were approximately 40% lower in GS967-treated *Scn1a* ^+/−^ mice (*p* < *0.016*, Fig. 5a,b), but there was no difference between untreated and lamotrigine-treated mice (Fig. 5c,d). Transcript levels of *Scn8a* were not different between untreated and GS967-treated mice (Supplemental Fig. S5), suggesting that the molecular basis for reduced Na_V_1.6 protein is posttranscriptional. These findings suggest that chronic GS967 treatment reduces Na_V_1.6 protein levels, which correlates with the normalization of pyramidal neuron sodium current density and may contribute to the unexpected antiepileptic effect of the compound in *Scn1a* ^+/−^ mice.

## Discussion

Dravet syndrome patients are often refractory to treatment with conventional antiepileptic therapies, and seizures may be exacerbated by certain drugs acting through block of sodium channels. Due to the rare nature of Dravet syndrome, there have been few randomized controlled trials of new or existing antiepileptic drugs^24^. Only stiripentol is specifically approved as an add-on treatment for Dravet syndrome, although there is widespread use of other drugs including valproic acid, clobazam and topiramate^25, 26^. Thus, there is a pressing need for additional therapeutic options. In this study, we demonstrated the antiepileptic effect of GS967 in the *Scn1a* ^+/−^ mouse model of Dravet syndrome, an unexpected result given that GS967 is a sodium channel blocker.

Mutations in *SCN1A*, the most frequent genetic cause of Dravet syndrome, are hypothesized to result in loss of GABAergic inhibitory tone based on neurophysiological investigations of *Scn1a* ^+/−^ mice^13, 14, 27^. Consistent with this cellular mechanism is the efficacy of antiepileptic drugs that potentiate GABAergic neurotransmission and the aggravation of the disease by certain sodium channel inhibitors. However, reduced sodium current density and blunted excitability in GABAergic interneurons are observed in *Scn1a* ^+/−^ mice prior to the onset of epilepsy^13, 16^. This suggests that other neural mechanisms, which may represent aberrant adaptations to *Scn1a* haploinsufficiency, could also contribute to neuronal hyperexcitability and therefore might present therapeutic opportunities.

We previously reported evidence of age-dependent differences in sodium current density and spontaneous firing of acutely isolated excitatory neurons in Dravet syndrome mice^16^. Specifically, hippocampal pyramidal neurons from *Scn1a* ^+/−^ mice at an age when seizure frequency is highest (P21–24) exhibit elevated sodium current density, a hyperpolarizing shift in the voltage-dependence of activation and a propensity for high frequency spontaneous action potentials that are not observed in wildtype littermates or in younger (P14–16) *Scn1a* ^+/−^ mice. Conceivably, the combination of lower sodium current in GABAergic interneurons with enhanced sodium current in excitatory neurons creates a substantial inhibitory-excitatory imbalance that promotes seizures.

Initially, the anticonvulsant effect of GS967 in *Scn1a* ^+/−^ mice seemed counterintuitive because this compound was originally reported to have preferential effects on persistent sodium current with little block of peak transient current^17^. However, we recently discovered additional biophysical effects of this compound on heterologously expressed cardiac sodium channels^28^. Similarly in this study, we demonstrated significant effects of GS967 on neuronal sodium current including a potent stabilization of inactivation leading to strong use-dependent block (Supplemental Fig. S2). These properties resemble other antiepileptic drugs that act through a mechanism resembling local anesthetic agents, but the effects observed for GS967 in *Scn1a* ^+/−^ mice cannot be generalized to all sodium channel blockers as evidenced by the lack of anticonvulsant efficacy of lamotrigine.

Other more long-term effects of GS967 may be responsible for its anticonvulsant efficacy. Chronic GS967 treatment was associated with suppression of spontaneous action potential firing of pyramidal neurons (Fig. 3), normalization of both sodium current density and voltage-dependence of activation in these cells (Fig. 4) following removal of the compound. In parallel, bipolar neurons from chronically GS967-treated mice exhibited higher peak sodium current density. However, there were no differences in sodium current density elicited at voltages near the action potential threshold among bipolar neurons from untreated, GS967- and lamotrigine-treated mice (Fig. 4) and this provides an explanation for the absence of differences in action potential firing frequency (Fig. 3). By contrast, lamotrigine treatment was associated with no change in neuronal excitability despite a significant enhancement of sodium current density in pyramidal neurons. However, lamotrigine treatment did not induce a change in the voltage-dependence of activation (Supplemental Fig. S3), nor reduce sodium current elicited near action potential threshold (Fig. 4). We speculate that there may be a ‘ceiling’ effect in pyramidal neurons in which action potential firing is at maximum and further elevations of sodium current without a change in voltage-dependence of activation does not affect firing. Lamotrigine effects on brain targets other than Na channels might also contribute to the worsening of the seizure phenotype in *Scn1a* ^+/−^ mice^29, 30^. Although we may infer that network excitability has been dampened by GS967 given the overt suppression of seizures, we did not specifically investigate the effects of GS967 on intact neuronal networks such as in acute brain slices. Investigating neuronal network effects of GS967 or other sodium channel blockers such as lamotrigine will be the focus of future experiments.

We also observed that chronic GS967 treatment, but not lamotrigine exposure, is associated with a significant reduction of total hippocampal Na_V_1.6 protein levels (Fig. 5) without a corresponding effect on *Scn8a* mRNA expression (Supplemental Fig. S4). We speculate that lower Na_V_1.6 protein levels may be due to impaired trafficking by a mechanism similar to some K_V_11.1 (hERG) blockers that promote channel ubiquitination and degradation or by inhibiting forward trafficking from the endoplasmic reticulum^31^. However, it is plausible that changes in Na_V_1.6 protein levels are due to indirect effects of GS967, possibly a response to reduced seizure frequency. The mechanism by which GS967 treatment reduces Na_v_1.6 protein will need further exploration.

Several lines of evidence point to a secondary involvement of Na_V_1.6 in the pathogenesis of seizures in Dravet syndrome. Previous studies have shown a relationship between Na_V_1.6 expression and seizure susceptibility, and Na_v_1.6 expression is increased in the CA3 hippocampus of amygdala-kindled rats^32^. Recent human genetic studies have identified gain-of-function mutations in *SCN8A*, which encodes Na_v_1.6, as a cause of severe epileptic encephalopathy^33, 34^. Other studies with *Scn8a* mutant mice have demonstrated that impaired Na_V_1.6 function confers seizure protection. Specifically, two mutant *Scn8a* mouse alleles (*Scn8a* ^med-jo/+^ and *Scn8a* ^med/+^) conferring impaired Na_V_1.6 expression exhibit elevated thresholds to flurothyl- and kainate-induced seizures. Additionally, crosses with *Scn8a* ^med-jo/+^ and *Scn8a* ^med/+^ mice normalize flurothyl-induced seizure threshold and prolong survival of *Scn1a* ^+/−^ mice^35^. Further work has shown that deletion of *Scn8a* in adult mice protects against 6-Hz psychomotor seizures, increases latency to flurothyl- and kainate-induced seizures and reduces picrotoxin-induced seizure activity^36^. These studies indicating that reduced Na_v_1.6 function or expression confer seizure resistance in mice is consistent with our observation that pharmacologic reduction of Na_v_1.6 protein levels mediated by GS967 dampens seizure susceptibility in *Scn1a* ^+/−^ mice. In light of a recent report demonstrating the feasibility of generating subtype specific Na_V_ blockers, these findings collectively support the concept that selective Na_V_1.6 inhibition could be a promising therapeutic strategy in Dravet syndrome^37^.

## Methods

### Animals

All animal care and experimental procedures were performed in accordance with the National Institutes of Health Guide for the Care and Use of Laboratory Animals and were approved by the Northwestern University Institutional Animal Care and Use Committee. The principles outlined in the ARRIVE (Animal Research: Reporting of *in vivo* Experiments) guideline and Basel declaration (including the 3 R concept) were considered when planning experiments. Mice were group-housed in a pathogen free mouse facility under standard laboratory conditions (14-h light/10-h dark) and had access to food and water *ad libitum*, except during hyperthermia-induced seizure experiments. *Scn1a* ^+/−^ mice were generated as previously described and are maintained as a congenic line on the 129S6/SvEvTac (129.*Scn1a* ^+/−^) background^15^. For experiments, F1 generation mice were produced by crossing 129. *Scn1a* ^+/−^ mice with C57BL/6 J mice. The *Scn1a* genotype was determined as previously described^15^.

### Isolation of hippocampal neurons and electrophysiology

Electrophysiology experiments were performed on acutely dissociated hippocampal neurons isolated from untreated or treated *Scn1a* ^+/−^ mice. Hippocampal neurons were isolated as described previously^20^. The dentate gyrus was excluded from dissections. Pyramidal neurons were identified based on pyramidal shaped morphology and a long apical process as previously described^16^. Neurons having bipolar morphology, which we previously adjudicated as GABAergic interneurons by virtue of GAD67 expression^20^, were also selected for recording experiments. To exclude glia and oligodendrocytes, only cells with a resting membrane potential of −55 to −80 mV, which exhibited the ability to fire multiple action potential when stimulated were selected for recording. Smaller (capacitance <6pF) fusiform shaped neurons (presumed granular cells) were not selected for recording experiments. Whole-cell voltage clamp and current clamp recordings of neuronal cell bodies were performed as described previously^16, 20^. All voltage clamp recordings utilized a holding potential of −120 mV. Voltage and current clamp recordings were performed as previously described^38, 39^ Statistical comparisons were made using *Student’s* t-test or one-way ANOVA followed by Tukey’s post hoc and *p* < 0.05 was considered statistically significant.

### Survival analysis

At postnatal day 18 (P18), *Scn1a* ^+/−^ mice were weaned then randomly assigned to either GS967 or control treatment groups. Animals in the GS967 treatment group were provided chow containing GS967 (8 mg GS967/kg chow; dosage estimated as 1–1.5 mg/kg/day based on an assumed consumption of 3–4 g chow/day). Survival was monitored until 8 weeks of age. GS967 treatment was withdrawn at 8 weeks of age and survival was monitored until 12 weeks of age. Statistical comparisons were made using the Mantel-Cox log-rank test with GraphPad Prism 6.07 (La Jolla, CA, USA) and *p* < 0.05 was considered statistically significant.

### Evaluation of anticonvulsant activity in Scn1a^+/−^ mice

Anticonvulsant activity was evaluated by counting spontaneous seizures in untreated and drug-treated, *Scn1a* ^+/−^ mice. At P18, *Scn1a* ^+/−^ mice were randomly assigned to either GS967, lamotrigine or control treatment groups. GS967 and lamotrigine were administered orally through supplementation in chow. Lamotrigine chow was empirically formulated at an estimated dosage of 20 mg/kg/day (125 mg lamotrigine/kg chow), and this produced a plasma concentration of 3.5 μg/mL, which is within the human therapeutic range (2.6–15 μg/mL). Plasma and brain samples were isolated from experimental animals immediately following the treatment period. GS967 plasma and brain levels were determined using high performance liquid chromatograph−tandem mass spectrometry by Gilead Sciences (Foster City, CA). Lamotrigine plasma samples were assayed using a HPLC 9 Flexar Binary LC Pump Platform (Perkin-Elmer, Waltham, MA) equipped with a UV-vis detector and C18 column. The mobile phase consisted of acetonitrile: 20 mM ammonium acetate, pH 6.7 (30:70, v/v) with a flow rate of 0.5 ml/min and detection at a wavelength of 310 nm. Mice had access to control, GS967 or lamotrigine chow *ad libitum*. Anti-seizure activity was evaluated by counting the number of behavioral seizures (clonic convulsion with loss of posture) captured by video recording between age P22 and P24, which was the period of peak seizure incidence with minimal animal loss due to death, in treated and untreated animals. Digital video images were analyzed offline by an observer blinded to treatment. Statistical comparisons were made using non-parametric Mann-Whitney test with GraphPad Prism and *p* < 0.05 was considered statistically significant.

We performed a preliminary study to evaluate the validity of using video capture for seizure evaluation in *Scn1a* ^+/−^ mice. Behavioral seizures (generalized tonic-clonic seizures) were correlated with electroencephalographic seizures using video-electroencephalography (EEG) monitoring as previously described^20^. *Scn1a* ^+/−^ mice (P16) were implanted with prefabricated headmounts (Pinnacle Technology, Inc., Lawrence, KS, USA) and continuous video-EEG data were collected from P20 to P26. Data were acquired and analyzed with Sirenia software (Pinnacle Technology, Inc.) and electrographic seizure activity was scored manually. A second observer counted behavioral generalized tonic-clonic seizures using only the video record. A strong correlation between behavioral and electrographic seizures (κ = 1.0) was observed in untreated mice (2,688 hours, 25 mice). Video-EEG data were collected from a cohort of GS967-treated mice that began treatment at P18, had surgery to place EEG headmounts at P20 then underwent continuous video-EEG from P22-P26 (535 hours, 7 mice). Representative EEG recordings are illustrated in Supplemental Fig. S6. No difference was observed between behavioral and electrographic seizure frequencies in GS967-treated *Scn1a* ^+/−^ mice.

### Hyperthermia-induced seizures

Hyperthermia-induced seizures were examined in P14–16 *Scn1a* ^+/−^ mice. Prior to the induction of hyperthermia, GS967 was administered orally to lactating dams through supplementation in chow beginning at postnatal day 10. Previous experiments demonstrated that GS967 is efficiently transmitted from lactating dams to nursing pups. GS967 concentration in pooled plasma samples from P14 mice was 1.39 μM, and average concentration in brain homogenates from these animals was 2.46 ± 0.17 μM. Mouse core body temperature was controlled by a rodent temperature regulator (TCAT-2DF, Physitemp Instruments, Inc, Clifton, NJ, USA) reconfigured with a Partlow 1160+ controller (West Control Solutions, Brighton, UK) connected to a heat lamp and RET-3 rectal temperature probe. Mice acclimated to the temperature probe for 5 minutes prior to induction of the hyperthermia protocol. Mouse core body temperature was elevated 0.5 °C every two minutes until the onset of the first clonic convulsion with loss of posture or until 42.5 °C was reached. Mice that reached 42.5 °C were held at temperature for 3 minutes. If no seizure occurred, the experiment was terminated and the mouse was considered seizure-free. Statistical comparisons were made using Mantel-Cox log-rank test with GraphPad Prism 6.07 and *p* < 0.05 was considered statistically significant.

### Hippocampal sodium channel expression

At postnatal day 18 (P18), *Scn1a* ^+/−^ mice were randomly assigned to either GS967 or control treatment groups. Following 5 days of treatment, hippocampi were dissected from P23 mice at and prepared for protein or transcript analysis. Separate cohorts of mice were used for protein and transcript analysis.

Western blot analysis was performed on hippocampal membrane proteins that were isolated by differential centrifugation from P23 untreated, GS967-treated or lamotrigine-treated *Scn1a* ^+/−^ mice. Membrane proteins were separated on a 7.5% SDS-PAGE gel and transferred to a PVDF membrane. Proteins were detected with primary antibodies directed against Na_v_1.1 (mouse; anti-Na_v_1.1 clone K74/71; 1:200, Neuromab, Davis, CA, USA), Na_v_1.2 (rabbit; anti-Na_v_1.2 polyclonal; 1:500; Alomone Labs, Jerusalem, Israel), Na_v_1.6 (rabbit; anti-Na_v_1.6 polyclonal; 1:500; Alomone Labs) or loading controls (mouse anti-β-tubulin clone TUB2.1, 1:5000; Sigma-Aldrich, St. Louis, MO, USA; mouse anti-mortalin, lot# 75–127, 1:1000; NeuroMab). Immunoreactive bands were detected on an Odyssey imager using fluorescent secondary antibodies directed at the primary antibodies (goat:anti-rabbit 800 or anti-mouse 680; 1:20,000; Thermo Scientific, Waltham, MA, USA). Densitometry was performed and band intensity of sodium channels were normalized to that of β-tubulin or mortalin used as loading controls. Statistical comparisons were made using Student’s t-test and *p* < 0.05 was considered statistically significant.

Steady-state mRNA levels for *Scn8a* were assessed by digital droplet RT-PCR. Hippocampal total RNA was extracted from P23 GS967-treated and untreated mice using TRIzol reagent according to the manufacturer’s instructions. First-strand cDNA was synthesized from 2 µg of oligo(dT) primed total RNA using Superscript III reverse transcriptase according to the manufacturer’s instructions (Life Technologies). First-strand cDNA samples were diluted 1:5 and 5 µl was used as template. Quantitative digital droplet PCR (ddPCR) was performed using ddPCR Supermix for Probes (No dUTP) (Bio-Rad, Hercules, CA, USA) and TaqMan Gene Expression Assays (Life Technologies) for mouse *Scn8a* (FAM-MGB-Mm00488110_m1) and *Tbp* (VIC-MGB-Mm00446971_m1). Reactions were partitioned into 20,000 droplets (1 nL each) in a QX200 droplet generator (Bio-Rad). Thermocycling conditions were 95 °C for 10 minutes, then 40 cycles of 95 °C for 15 seconds and 60 °C for 1 minute (ramp rate of 2 °C/sec) and a final inactivation step of 98 °C for 10 minutes. Following amplification, droplets were analyzed with a QX200 droplet reader with QuantaSoft v1.6.6.0320 software (Bio-Rad). All assays lacked detectable signal in no-RT and no template controls (data not shown). Relative transcript levels are expressed as a ratio of the *Scn8a* concentration to *Tbp* concentration. Statistical comparison between groups was made using *Student’s* t-test and *p* < 0.05 was considered statistically significant.

## Electronic supplementary material

Supplemental Information
