# Supplementary material for: Unexpected Efficacy of a Novel Sodium Channel Modulator in Dravet Syndrome
Source: Sci Rep. 2017 May 10;7:1682. doi: 10.1038/s41598-017-01851-9 (PMC5431801; doi:10.1038/s41598-017-01851-9)
Supplement: Supplementary file 1 — Supplemental Information [file 41598_2017_1851_MOESM1_ESM.pdf]

Unexpected Efficacy of a Novel Sodium Channel Modulator in Dravet Syndrome

Lyndsey L. Anderson, Nicole A Hawkins, Christopher H. Thompson,

Jennifer A. Kearney and Alfred L. George, Jr.

EXTENDED DATA FIGURES

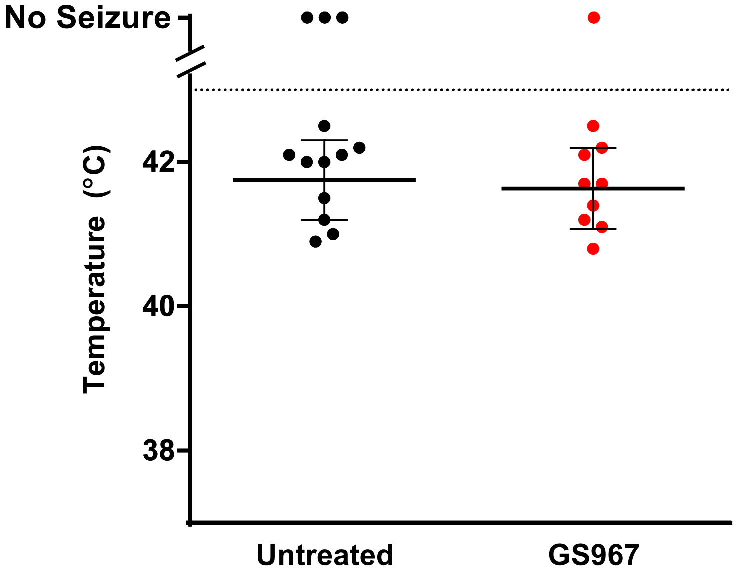

Supplemental Figure S1 | GS967 was not effective at preventing hyperthermia-induced seizures in *Scn1a*$^{+/-}$ mice. Temperature of generalized tonic-clonic seizure for individual untreated or GS967-treated mice in response to a thermal challenge. The average temperatures of seizure induction are depicted by the thick black line. Error bars represent SEM, with n = 9-10 per group. There is no statistical difference between groups.

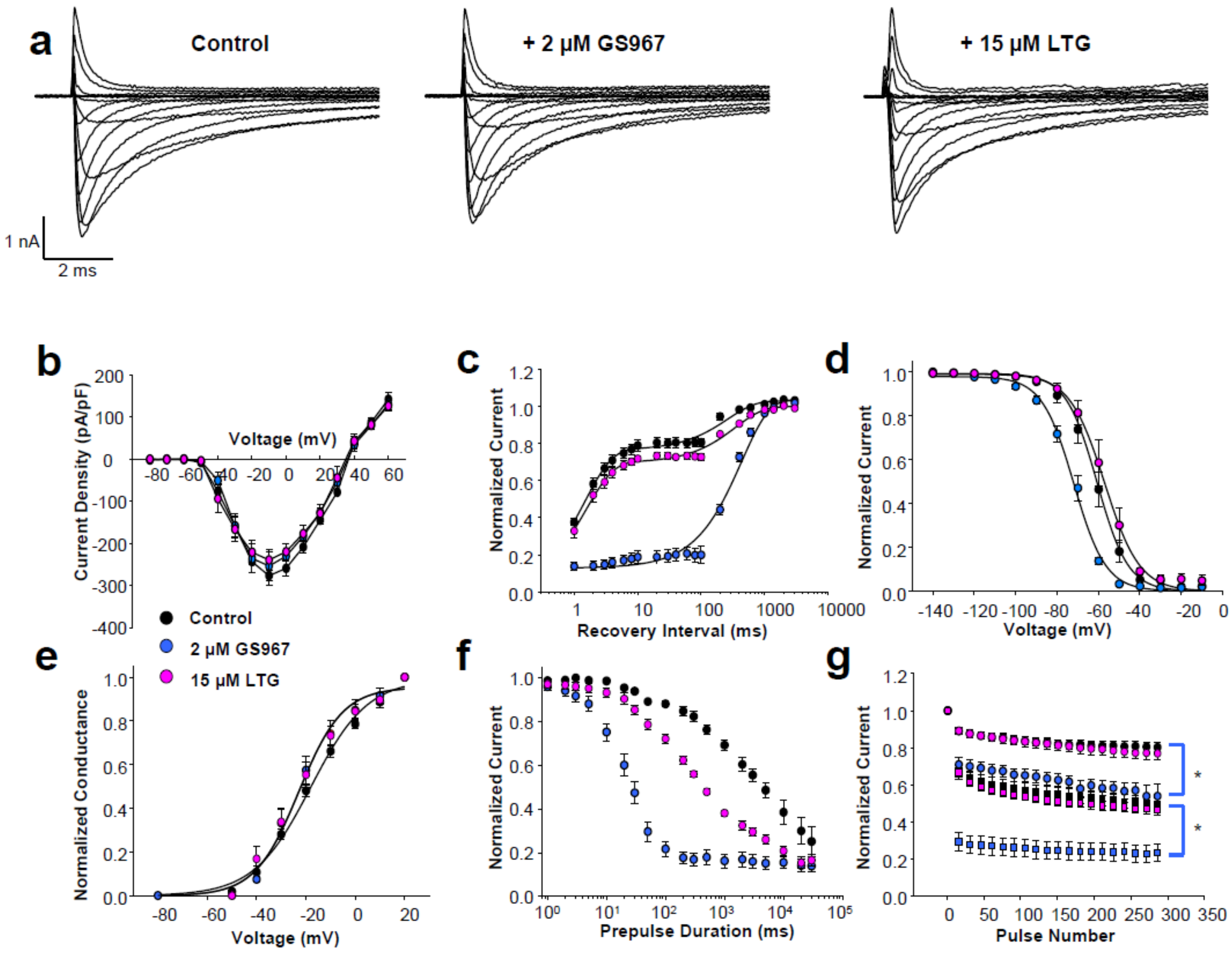

Supplemental Figure S2 | GS967 inhibits peak current and stabilizes channel inactivation of voltage-gated sodium channels in hippocampal pyramidal neurons. (**a**) Representative traces of sodium current in the absence (*left*) or presence either 2 μM GS967 (*middle*) or 15 μM lamotrigine (LTG, *right*). (**b**) Current-voltage relationship of neuronal voltage-gated sodium current in the presence or absence of GS967 or LTG. (**c**) Recovery from fast inactivation in the absence or presence of GS967 or LTG. (**d**) Steady-state channel inactivation in the absence or presence of GS967 or LTG. The $V_{½}$ values derived from Boltzmann fits were -58.1 ± 3.1 mV (n = 8), - 71.5 ± 2.1 mV (n = 8) and -57.4 ± 3.5 mV (n = 5) for control, GS967-treated and LTG-treated neurons, respectively. $V_{½}$ for GS967-treated neurons was significantly different from control (p=0.013) and LTG-treated (p=0.003). (**e**) Voltage-dependence of activation in the presence or absence of GS967 or Lamotrigine. (**f**) Onset of slow inactivation in the presence or absence of GS967 or LTG. (**g**) Use-dependent block in response to a 5 ms step to 0 mV from a holding potential of -120 mV at frequencies of 10 Hz (circles) or 100 Hz (squares) in the presence or absence of GS967 or LTG (**p* < 0.001 comparing control with GS967; Student's t-test). All data are expressed as mean ± SEM, with n = 5 -15 for each condition

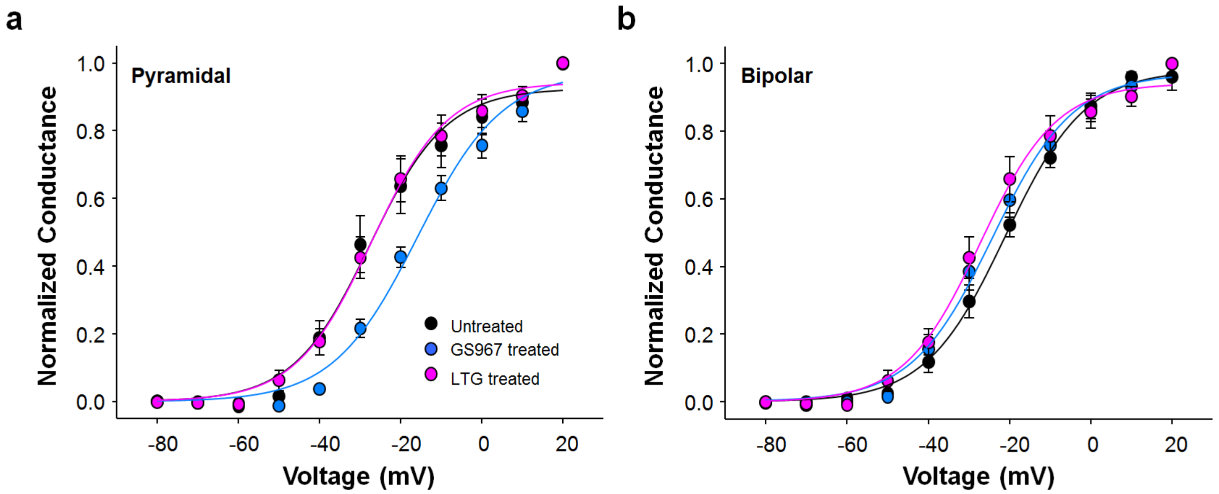

Supplemental Figure S3 | Voltage-dependence of activation for neuronal sodium current. Conductance-voltage relationships for voltage-dependent sodium current from **(a)** pyramidal neurons and **(b)** bipolar neurons isolated from untreated (black symbols), GS967-treated (blue symbols), or LTG-treated (purple symbols) *Scn1a*$^{+/-}$ mice. All data are expressed as mean ± SEM for n = 7 – 16 cells. Boltzmann fits to the data produced the following parameters:

Pyramidal neurons	**$V_{1/2}$ (mV)**	**Slope factor (k)**	**n**
Untreated	-27.5 ± 3.3	8.6 ± 1.3	10
GS967-treated	-18.4 ± 1.0*	8.9 ± 0.4	16
LTG-treated	-26.3 ± 2.6	9.9 ± 1.9	7

** p=0.029 (untreated vs GS967); * p=0.038 (GS967 vs LTG)*

Bipolar neurons

Untreated	-21.0 ± 1.7	9.1 ± 1.0	7
GS967-treated	-23.6 ± 2.6	9.5 ± 0.6	9
LTG-treated	-24.6 ± 1.7	10.1 ± 1.7	7

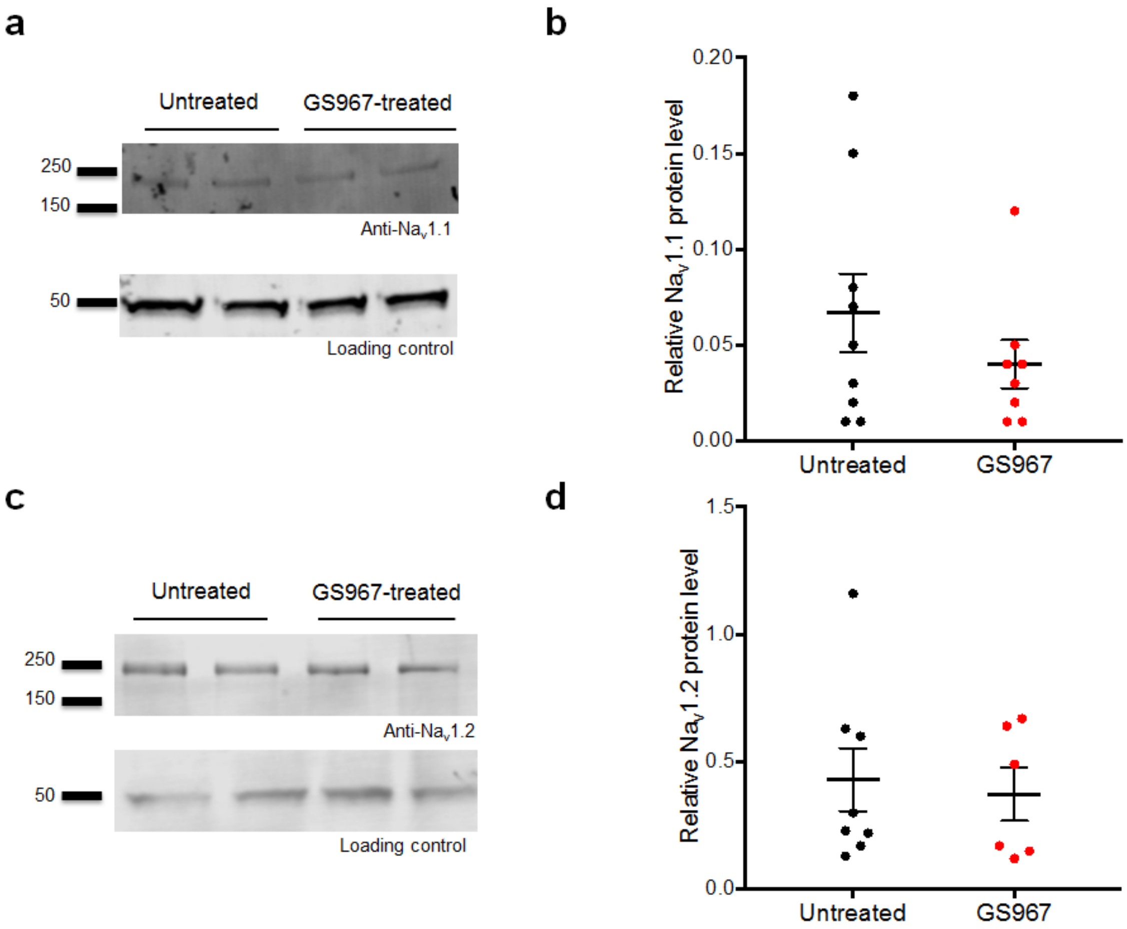

Supplemental Figure S4 | GS967 has no effect on neuronal Na_v1.1 and Na_v1.2 expression in *Scn1a*$^{+/-}$ mice.

(**a**) Western blot analysis of Na_v1.1 protein levels in hippocampal membrane preparations from *Scn1a*$^{+/-}$ mice. Representative blots of two biological replicates are shown for untreated and GS967-treated mice. (**b**) Scatter plot of densitometric analysis of Western blot data. Data points represent the individual densitometry for untreated or GS967-treated mice. The average densitometry values are depicted by the thick black line. Error bars represent SEM, with n=8-9 mice per treatment. There is no statistical difference between groups. (**c**) Western blot analysis of Nav1.2 protein levels in hippocampal membrane preparations from *Scn1a*$^{+/-}$ mice. Representative blot from two biological replicates are shown for untreated and GS967-treated mice. (**d**) Scatter plot of densitometric analysis of Western blot data. Data points represent the individual densitometry for untreated or GS967-treated mice. The average densitometry values are depicted by the thick black line. Error bars represent SEM, with n=6-8 per treatment. There is no statistical difference between groups.

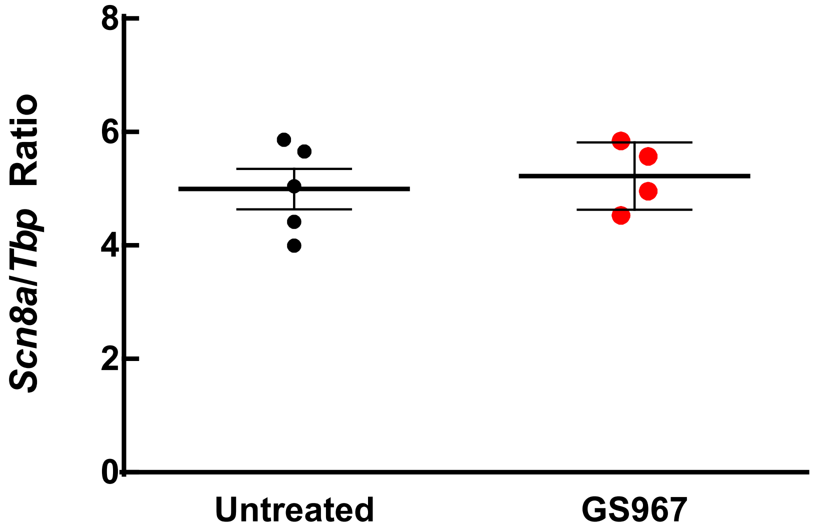

Supplemental Figure S5 | Comparison of *Scn8a* transcript expression in untreated and GS967-treated *Scn1a*$^{+/-}$ mice.

Data represent relative hippocampal transcript levels determined by ddPCR. Data are expressed as a ratio of *Scn8a* to *Tbp*. Data represent the average *Scn8a*/*Tbp* ratio (≥ 2 technical replicates) for individual untreated or GS967-treated mice. The average *Scn8a*/*Tbp* ratios are depicted by the thick black line. Error bars represent SEM, with n = 4-5 mice per group. There is no statistical difference between groups.

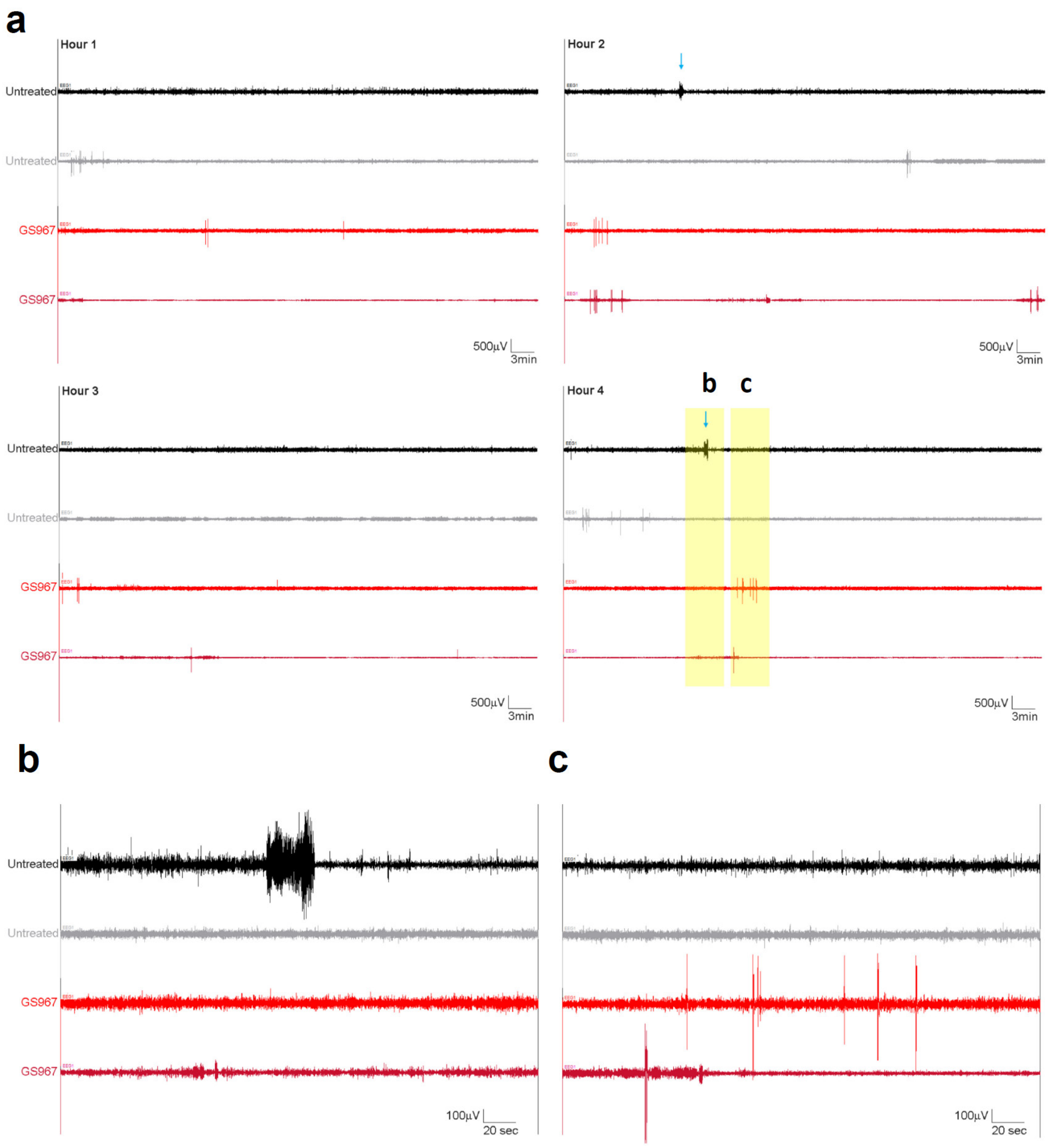

Supplemental Figure S6 | Representative EEG records from untreated and GS967-treated *Scn1a*$^{+/-}$ mice.

(**a**) Four continuous hours of EEG from one channel is displayed for two untreated and two GS967-treated *Scn1a*$^{+/-}$ mice. Blue arrows mark GTC seizures. (**b,c**) Zoomed images of the yellow shaded segments from (**a**) showing a representative GTC (**b**) and high amplitude non-ictal events (**c**).

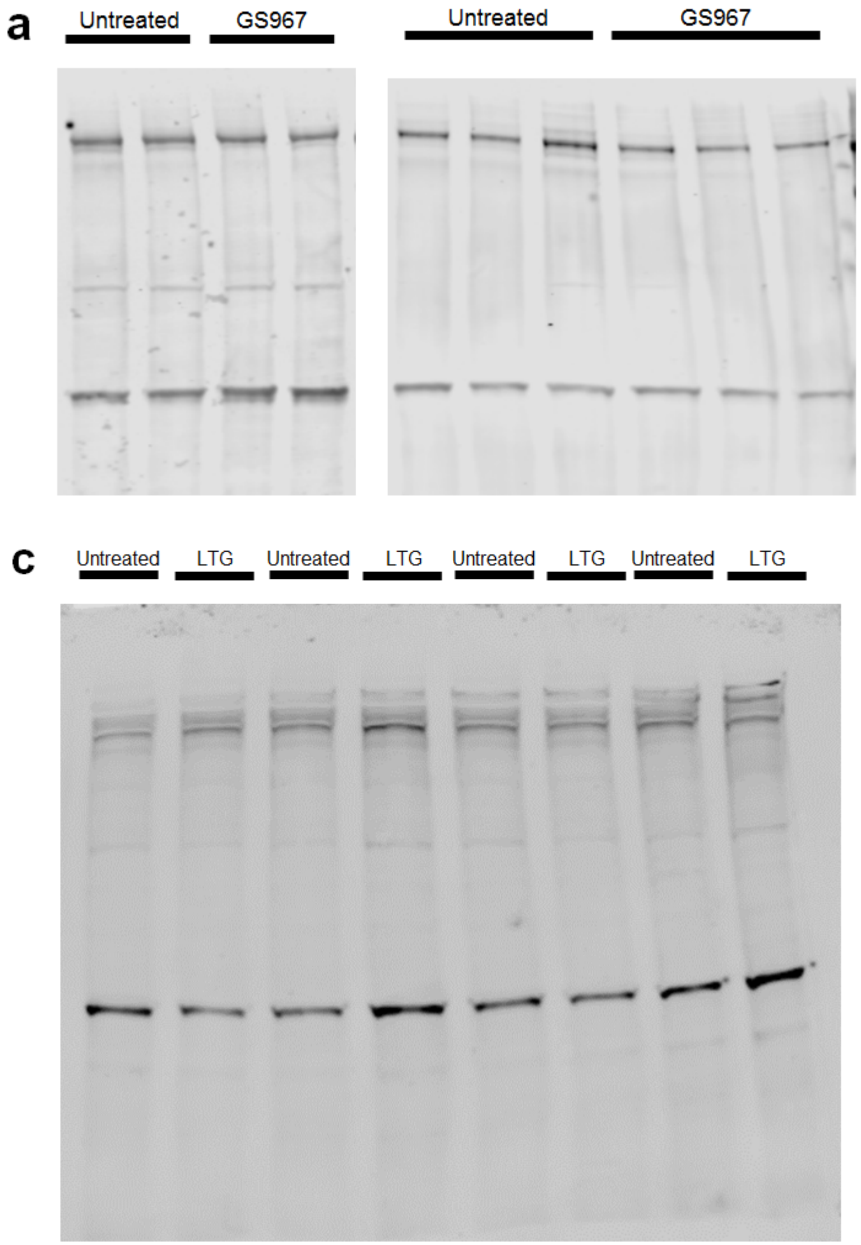

Supplemental Figure S7 | Full-length western blot images corresponding to Fig. 5.
